# Proteome and Ubiquitylome Analyses of Maize Endoplasmic Reticulum under Heat Stress

**DOI:** 10.3390/genes14030749

**Published:** 2023-03-19

**Authors:** Chunyan Gao, Xiaohui Peng, Luoying Zhang, Qi Zhao, Liguo Ma, Qi Yu, Xuechun Lian, Lei Gao, Langyu Xiong, Shengben Li

**Affiliations:** 1 State Key Laboratory of Crop Genetics & Germplasm Enhancement and Utilization, College of Life Sciences, Nanjing Agricultural University, Nanjing 210095, China; 2 College of Agriculture, Nanjing Agricultural University, Nanjing 210095, China; 3 College of Life Sciences and Oceanography, Shenzhen University, Shenzhen 518060, China; 4 Guangdong Provincial Key Laboratory for Plant Epigenetics, College of Life Sciences and Oceanography, Shenzhen University, Shenzhen 518060, China; 5 Institute of Advanced Studies in Humanities and Social Sciences, Beijing Normal University, Zhuhai 519087, China; 6 Academy for Advanced Interdisciplinary Studies, Nanjing Agricultural University, Nanjing 210095, China

**Keywords:** heat stress, endoplasmic reticulum, proteomics, ubiquitination, maize

## Abstract

High temperatures severely affect plant growth and pose a threat to global crop production. Heat causes the accumulation of misfolded proteins in the endoplasmic reticulum(ER), as well as triggering the heat-shock response (HSR) in the cytosol and the unfolded protein response (UPR) in the ER. Excessive misfolded proteins undergo further degradation through ER-associated degradation (ERAD). Although much research on the plant heat stress response has been conducted, the regulation of ER-localized proteins has not been well-studied thus far. We isolated the microsome fraction from heat-treated and untreated maize seedlings and performed proteome and ubiquitylome analyses. Of the 8306 total proteins detected in the proteomics analysis, 1675 proteins were significantly up-regulated and 708 proteins were significantly down-regulated. Global ubiquitination analysis revealed 1780 proteins with at least one ubiquitination site. Motif analysis revealed that alanine and glycine are the preferred amino acids upstream and downstream of ubiquitinated lysine sites. ERAD components were found to be hyper-ubiquitinated after heat treatment, implying the feedback regulation of ERAD activity through protein degradation.

## 1. Introduction

An appropriate range of temperature is very important for plant growth. As such, the gradual increase in temperatures worldwide poses a severe threat to agriculture. High temperatures affect plant growth in multiple aspects, including disordered protein conformation, reduced enzyme activity, change of membrane permeability, and unbalanced redox homeostasis [1,2]. Generally, nascent peptides must undergo necessary folding to attain an appropriate conformation before reaching their final destination in the cell [3]. A prominent consequence of heat stress is the accumulation of incorrectly folded proteins, resulting in ER stress [4]. Plants have evolved sophisticated mechanisms to alleviate the damage caused by these misfolded proteins. 

In the cytosol, a well-established strategy is the heat shock response, in which a set of heat-shock proteins (HSPs) are rapidly induced in response to high temperatures [5,6]. HSP protein families are conserved in procaryotes and eucaryotes, and are characterized by the heat-shock domain at the C-terminus. According to their molecular weights, HSPs are divided into five groups: HSP100, HSP90, HSP70, HSP60, and small heat-shock proteins (sHSPs) [7,8]. These HSPs serve as chaperone molecules to prevent protein aggregation, further facilitating protein folding and transport [9,10]. Proteomics studies revealed that maize HSP26, HSP16.9, and other HSPs were rapidly up-regulated in response to high temperatures [11]. The induction of HSPs by HSR is largely attributed to the mobilization of heat-shock factors (HSFs) [12]. HSFs are a group of highly conserved proteins, categorized into A, B, and C classes. All HSFs contain a DNA binding domain at the N-terminus, which is necessary for the binding with heat-shock elements (HSEs) in the promoter of HSP genes [13]; however, only class A members have an activation domain at the C-terminus, which is required for transcription activation. Class B and C members lack this domain and are unable to activate HSP gene transcription on their own [14,15]. Under normal conditions, class A1 HSFs are associated with HSP70/90 and their co-chaperones. When plants are challenged with heat stress, these chaperones are recruited by excessive misfolded proteins, and the released HSFs move into the nucleus to trigger the transcription of HSP genes [16,17,18]. The maize genome has been predicted to encode 31 HSFs, among which *ZmHsf13*, *ZmHsf04*, *ZmHsf23*, *ZmHsf11,* and *ZmHsf25* have been shown to be significantly induced by high-temperature treatment [19]. 

In the endoplasmic reticulum, the accumulation of misfolded proteins caused by heat and other stressors triggers the unfolded protein response (UPR) [20]. Two pathways involved in the UPR have been discovered. One pathway relies on peptide cleavage of the transcription factor bZIP28, which contains a bZIP domain at the N-terminus and a transmembrane domain (TMD) at the C-terminus, separated by a protease recognition motif [21]. Under normal conditions, bZIP28 is translated into a full-length protein with the TMD domain and is anchored on the ER. Heat or other stresses trigger cleavage of this protein, releasing the N-terminal fragment from the ER to the nucleus and promoting the transcription of UPR genes [21,22]. The second pathway of the UPR is dependent on the alternative splicing of another transcription factor gene, *bZIP60*. Under normal conditions, the *bZIP60* transcript encodes a protein with a bZIP domain followed by a TMD domain and localizes to the ER. Under heat or other stress conditions, an ER-localized kinase/endoribonuclease, IRE, triggers the alternative splicing of *bZIP60* RNA, which results in a frame shift after the bZIP domain. The encoded protein loses the TMD domain, creating a nucleus localization signal which brings the protein into the nucleus to activate the transcription of UPR genes [23,24,25].

In addition to HSR and UPR, heat also induces ER stress, which can be alleviated by ER-associated degradation (ERAD). In the ER, protein folding is monitored by the ER-associated protein quality control (ERQC) system. Well-folded and misfolded proteins can be distinguished according to various polysaccharide modifications [26]. Typically, misfolded proteins are recognized by the ERQC system and undergo several rounds of re-folding. If proteins fail to be shaped into the correct conformation, they will be labeled with ubiquitin and retro-translocated from the ER to the cytosol for degradation by the 26S proteasome [27,28]. Many ERAD components have been identified through genetic and biochemical studies in plants, and have been found to share a highly conserved mechanism in yeast and animals [29,30,31]. 

Transcriptome and proteome analyses have revealed several genes responsive to heat stress in maize [11,32,33]. The ER plays a central role in sensing the heat stress signal and triggering the HSR, UPR, and ERAD responses. Protein localization and modification in the ER are dynamic and finely controlled in cells. However, proteomics and ubiquitylome investigations based on total protein extracts may not precisely mirror the protein situation in the ER. To better understand the changes in ER protein abundance and ubiquitination status during heat stress, we isolated microsomes from maize seedlings with and without heat stress, then conducted protein quantification and ubiquitylome analyses. A total of 2383 differentially expressed proteins were discovered through proteomics analysis, and 1780 proteins were identified as ubiquitination targets. As a protein degradation system, the ERAD pathway is determined to be under feedback regulation through protein ubiquitination modification.

## 2. Materials and Methods

### 2.1. Plant Materials and Heat Treatment

Maize inbred line KN5585 plants were grown in a growth chamber at 28 °C with a photoperiod of 14 h light and 10 h dark at 60% relative humidity. For heat treatment, two-week old seedlings were transferred to a chamber pre-heated to 42 ± 1 °C for 1 h [19]. After treatment, shoot tissues were collected and stored at −80 °C for RNA and microsome isolation. 

### 2.2. QRT-PCR and RNASeq 

Maize tissue was ground into a fine powder in liquid nitrogen and 100 mg of powder was used for RNA isolation with TRIzol reagent (Invitrogen, Waltham, MA, USA). A quantity of 1 μg of total RNA was used to synthesize the first strand of cDNA using EasyScript^®^ All-in-One First-Strand cDNA Synthesis SuperMix for qPCR (Transgen, Beijing, China), according to the manufacturer’s instructions. Fluorescent quantitative PCR was performed using ChamQ SYBR qPCR Master Mix (Vazyme, Nanjing China). Relative gene expression was calculated using the 2^−ΔΔCt^ method, and the maize *TUBULIN* gene (*Zm00001d046996*) served as the internal control. The primers used in this study are presented in Supplementary Table S1.

To construct the RNAseq library, mRNAs were purified by Oligo (dT) magnetic beads and degraded into 300 nt fragments with RNA fragmentation reagent (Ambion, Austin, TX, USA). cDNAs were synthesized by reverse transcription with random hexamer primers. RNAseq libraries were generated using an NEBNext Ultra RNA library prep kit (NEB, Ipswich, MA, USA), and 150 bp pair-end RNA-sequencing was conducted using the Illumina NovaSeq 6000 platform at Personalbio company. The significance of differentially expressed genes (DEGs) was determined by fold change ≥ 2 and *p*-value < 0.05. Tree biological replicates were performed for qPCR and RNAseq, and at least 10 seedlings were combined to isolate RNA for each replicate. 

### 2.3. Microsome Isolation

Microsome isolation was performed as previously described [34]. Briefly, 10–15 g seedlings were ground in liquid nitrogen and re-suspended in 20 mL microsome extraction buffer (MEB; 100 mM Tris-Cl, pH 7.5, 5 mM EGTA, 15 mM MgCl_2_, 5 mM DTT, 0.3 M sucrose, 50 mg/L cycloheximide, 50 mg/L chloramphenicol, and a complete proteinase inhibitor cocktail, MedChemExpress, Shanghai, China). The cell lysate was filtered with two layers of miracloth, then centrifuged twice at 10,000× *g* for 10 min at 4 °C to remove debris. Then, 100 μL of supernatant was taken for protein input, and the rest of the supernatant was loaded onto a sucrose step gradient (2.5 mL 0.6 M/2.5 mL 1.7 M sucrose) and centrifuged at 28,000 rpm at 4 °C in a Beckman SW41 rotor for 1 h. The interface of the two gradients was transferred to a new tube and diluted with 10 × volume of MEB. The microsome was pelleted by centrifugation at 28,000 rpm for 0.5 h, followed by washing twice with the same buffer. Finally, the microsome was resolved in protein extract buffer containing 6 M guanidine, 10 mM DTT, and 20 mM Tris (pH 8.0).

### 2.4. Western Blot Analysis

Maize proteins were isolated and detected according to a previously published protocol [35]. Total and microsome proteins were separated using 10% SDS-PAGE and blotted onto polyvinylidene difluoride (PVDF) membranes (Bio-Rad, Hercules, CA, USA). Mouse antibodies against plant HSC70, HSP90, and NADPH4 were used at ratios of 1:2000, 1:10,000, and 1:5000, respectively. 

### 2.5. Sample Preparation for iTRAQ and Ubiquitylome Analysis 

For the iTRAQ experiment, microsome proteins were treated with 20 mM DTT at 60 °C for 30 min. After cooling to room temperature, 40 mM iodoacetamide was added to the samples, which were kept in darkness for 1 h to block free cysteine. After that, 2 M urea and 50 mM NH_4_HCO_3_ were used to dialyze the samples twice, then 50 mM NH_4_HCO_3_ alone for 1.5 h twice. Finally, trypsin was added to the samples at a ratio of 1:50 (*w*:*w*) and incubated at 37 °C overnight. The digested peptides were filtered with Amicon ultra centrifugal filters, and the residual primary amine from the reagent was extensively removed by vacuum. For iTRAQ, 100 µg of digested peptides were labeled using a Thermo TMT10 plex (Lot #: WC318970) following the manufacturer’s protocol. Labeled samples were mixed equally and analyzed by LC-MS/MS to validate the labeling efficiency and normalization factor. The pooled samples were dried and desalted with SPEC Pt C18 (Agilent Technologies, Santa Clara, CA, A57203, USA). For the ubiquitylome analysis, the tryptic digested samples were subjected to immuno-affinity purification (IAP) using a PTMScan^®^ Ubiquitin Remnant Motif (K-ε-GG) Kit (Cell Signaling #5562). 

### 2.6. Nano LC-MS/MS Analysis

Nano LC-MS/MS was performed using a Dionex rapid-separation liquid chromatography system interfaced with an Eclipse (Thermo Fisher Scientific, Waltham, MA, USA). The peptide fractions or K-ε-GG-enriched peptides were loaded onto an Acclaim PepMap 100 trap column (75 µm × 2 cm; Thermo Fisher) and washed with buffer A (0.1% trifluoroacetic acid) for 5 min at a flow rate of 5 µL/min. The trap was brought in-line with the nano analytical column (nanoEase, MZ peptide BEH C18, 130 A, 1.7 µm, 75 µm × 20 cm, Waters) with flow rate of 300 nL/min and eluted by a multi-step gradient (4% to 15% buffer B containing 0.16% formic acid and 80% acetonitrile for 20 min, then 15–25% buffer B for 40 min, followed by 25–50% buffer B for 30 min). The sequence scan began with an MS1 spectrum (Orbitrap analysis; resolution, 120,000; scan range, 375–1600 Th; automatic gain control target, 6E4; maximum injection time, 50 ms). The top S (3 s) duty cycle scheme was used to determine the number of parent ions investigated for each cycle. For proteomic samples without IAP, the SPS method was used. All data were analyzed using Maxquant and the Andromeda search engine. LC-MS data were searched against the uniprot maize database. The output result was further processed by Perseus (V1.6.5) for data polishing and statistical analysis. Protein or ubiquitination sites with FDR < 1% were represented. A fold change of ≥1.5 or <0.67 and a *t*-test *p*-value of < 0.05 were considered to indicate significantly changed proteins or PTM (post-translational modification) sites.

## 3. Results

### 3.1. Heat Treatment-Induced HSR Gene Expressions and Microsomes Were Enriched for ER 

**Figure 1:**
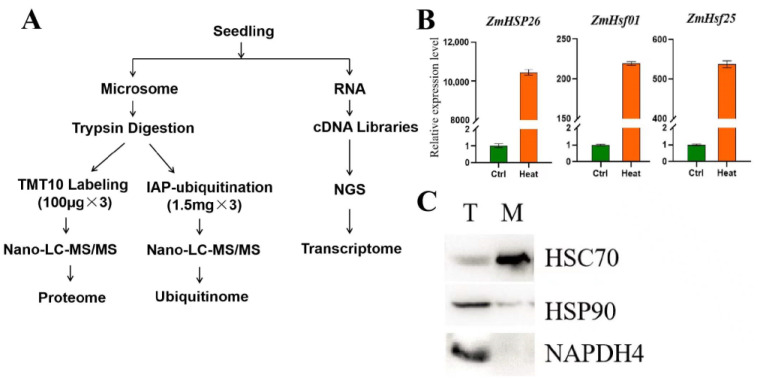
Experimental design and sample treatment: (**A**) Workflow for the experiment. Maize seedlings were subjected to heat stress. Total RNAs were used for RNAseq and microsomes were applied for proteome and ubiquitylome analyses. NGS, Next-generation sequencing; LC-MS/MS, liquid chromatography–tandem mass spectrometry; IAP, immuno-affinity purification; (**B**) qRT-PCR for maize HSR genes; and (**C**) purity of microsome fraction. HSC70, representative endoplasmic reticulum protein; HSP90, representative cytosol protein; NAPDH4, representative mitochondrion protein; T, total protein; M, microsome protein.

In order to investigate the changes in ER proteins under heat stress, we isolated microsomes and extracted proteins from maize for proteomic and ubiquitylome analyses (Figure 1A). Heat-shock responses are well-conserved mechanisms in plants. To assess whether our treatment triggered the anticipated responses, we first checked the expression levels of several known heat-shock responsive genes after heat treatment. We found that the *ZmHSP26*, *ZmHsf01,* and *ZmHsf25* genes were significantly induced by heat treatment (Figure 1B). In the microsome fraction, HSP70—an ER localized protein—was accumulated to a much higher level, compared to the total protein extract, while the cytosolic protein HSP90 and mitochondrial protein NADPH4 were almost depleted from the microsome fraction. This indicates that the microsome fraction was highly enriched for the ER (Figure 1C). 

### 3.2. Global Alterations in Transcription by Heat Treatment

**Figure 2:**
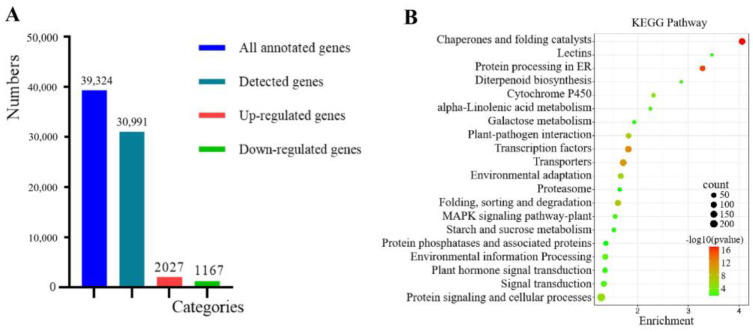
RNAseq analyses for the heat-treated maize seedlings: (**A**) The number of annotated and RNAseq-detected genes; and (**B**) KEGG analysis results for all up-regulated genes.

A total of 30,189 RNAs (76.8% of the annotated maize coding genes) were detected in our RNAseq data, and 3194 differentially expressed genes (DEGs) were identified. Among them, 2027 were up-regulated and 1167 were down-regulated (Figure 2A). KEGG (Kyoto Encyclopedia of Genes and Genomes) analysis implied that genes involved in chaperone and folding catalyst, protein processing in the endoplasmic reticulum, transcription factors, transporters, folding, sorting, and degradation pathways were significantly enriched in the DEGs (Figure 2B). Heat initiated HSR in the cytosol and elicited UPR in the endoplasmic reticulum. These responses up-regulated the expression of a constellation of genes to attenuate the damage from heat stress. Many genes in these pathways were up-regulated in our results. These included genes involved in the recognition of well-folded or misfolded proteins, such as the ER membrane chaperone *CNX* (*Zm00001d025305* and *Zm00001d003857*) and ER lumen chaperone *CRT* (*Zm00001d005460* and *Zm00001d012170*). Other chaperone genes involved in this process included the luminal chaperones *NEF* (*Zm00001d017809*), *BIP* (*Zm00001d014993*), *HSP40* (*Zm00001d009783* and *Zm00001d037586*), *GRP94* (*Zm00001d020827* and *Zm00001d041719*), and *PDI* (*Zm00001d049099*, *Zm00001d020687,* and *Zm00001d005866*), which break and rebuild the disulfide bonds to ensure proper folding and avoid protein aggregation. Besides UPR genes, several ERAD genes—including ubiquitin ligase complex genes—were also up-regulated. Three *Derlin* genes (*Zm00001d034871*, *Zm00001d037687,* and *Zm00001d010368*), which promote protein retro-translocation from the ER to the cytosol, and one ER-localized E3 ligase gene, *RMA1* (*Zm00001d024520*), were significantly up-regulated. Two *AAA-ATPase* genes (*Zm00001d014124* and *Zm00001d032859*) and a ubiquitin-like domain gene *DSK2* (*Zm00001d027514*), which transports ubiquitinated proteins to the proteasome, were over-accumulated. A large number of HSP genes and genes encoding the E2 ubiquitin conjugating enzyme were also highly induced by heat.

**Figure 3:**
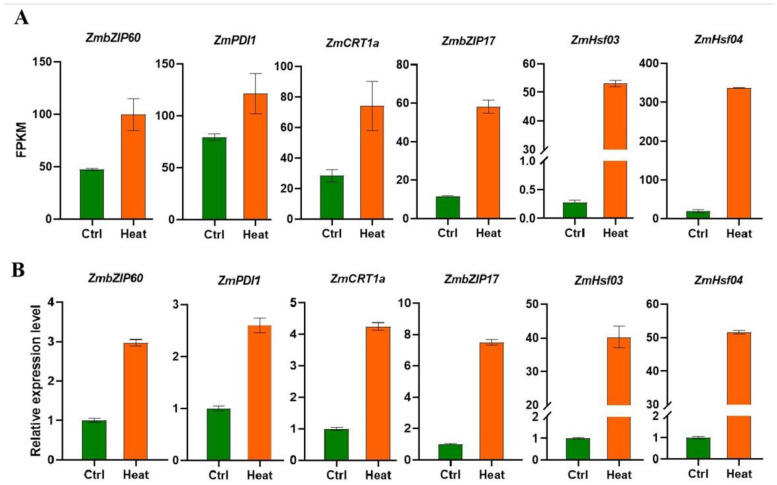
Validation of DEGs by quantitative real-time PCR: (**A**) FPKM values of six genes from RNAseq data; and (**B**) qRT-PCR validation of gene expression.

To confirm the expression changes detected in our RNAseq data, we chose several HSR genes for validation using real-time PCR. As the key transcription factors of HSP genes, *ZmHsf03* and *ZmHsf04* were verified to be promptly up-regulated after heat-shock treatment. Similarly, the expression of two important transcription factors in UPR—*ZmbZIP17* and *ZmbZIP60*—were also found to be highly induced by heat. In addition, other UPR genes, including the chaperone gene *CALRETICULIN1a* (*Zm00001d005460*) and the protein folding gene *PROTEIN DISULFIDE ISOMERASE1* (*Zm00001d049099*), were also significantly up-regulated after heat treatment (Figure 3).

### 3.3. Integrative Analysis of the ER Proteome and Transcriptome

**Figure 4:**
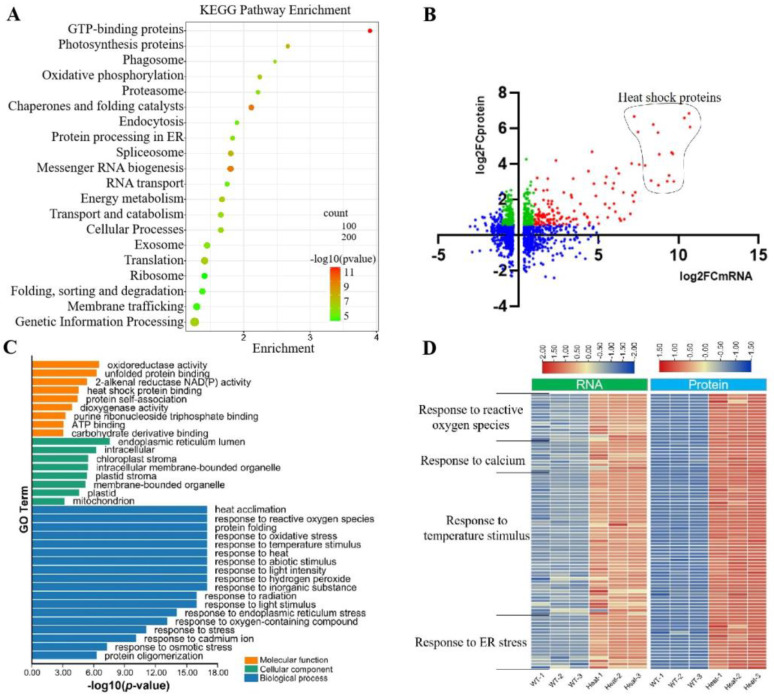
Integrative analyses of proteome and transcriptome on heat−treated maize: (**A**) KEGG analysis of the DEPs. Proteins are prepared from the ER; (**B**) coordination analysis of the proteomics and transcription data. The *x* and *y* axes represent the Log_2_FC of RNAseq and proteomics, respectively; (**C**) GO analysis of the genes simultaneously up−regulated at RNA and protein levels; and (**D**) heat maps of up−regulated DEGs for four major pathways.

To better understand the changes in protein accumulation on the ER after heat treatment, we isolated microsome fractions and performed iTRAQ analyses on maize seedlings with and without heat-shock treatment. A total of 8036 proteins were detected by mass spectrometry. Among them, 1675 and 708 proteins were significantly up-regulated and down-regulated, respectively. When analyzed for KEGG pathways, these differentially expressed proteins (DEPs) were found to be enriched in GTP-binding, chaperones and folding catalysts, messenger RNA biogenesis, spliceosome, photosynthesis proteins, oxidative phosphorylation, translation, energy metabolism, genetic information processing, and transport and catabolism pathways (Figure 4A). A Pearson’s correlation coefficient of r = 0.43 was observed between all significantly changed mRNAs and their cognate proteins. In total, 1412 differentially expressed genes (DEGs) and corresponding DEPs were matched. Of these, 143 genes were up-regulated at both the RNA and protein levels (Figure 4B, red). It is worth noting that 313 genes were accumulated only at the protein level, without changes in the RNA levels (Figure 4B, green).

We found that HSP genes were over-represented in the group of genes simultaneously up-regulated at both the RNA and protein levels (Figure 4B). HSPs were generally considered to be molecular chaperones involved in protein processing. The over-representation of HSP genes in this group suggests their central roles in responses to heat shock and other stresses. We conducted Gene Ontology (GO) analysis on these 143 genes. In the cellular component cluster, GO terms reflecting the endoplasmic reticulum lumen, chloroplast stroma, intracellular membrane-bounded organelle, plastid stroma, membrane-bounded organelle, and mitochondrion were enriched, consistent with the fact that our protein samples were derived from the microsome fraction. The enriched GO terms in terms of molecular function indicated that many DEGs and DEPs were involved in oxidoreductase activity, unfolded protein binding, 2-alkenal reductase NAD (P) activity, and heat-shock protein binding (Figure 4C). Excessive reactive oxygen species are produced in plants after heat shock, which can be utilized as signaling molecules to trigger defense responses. Previous studies have shown that HSFs act as H_2_O_2_ sensors and induce HSP gene expression. NADPH also affects the plant heat response, and a loss of NADPH oxidase reduced heat tolerance in *Arabidopsis* [36]. As shown in Figure 4D, 102 out of the 143 genes were mainly involved in the response pathways to ROS, calcium, temperature stimulus, and ER stress. 

### 3.4. Heat Treatment Changes the Protein Ubiquitination Status of Maize ER 

As one of the most common post-translational modifications, ubiquitylation is involved in nearly all physiological and signaling processes in plants, including hormone perception, photomorphogenesis, circadian rhythms, self-incompatibility, and defense against biotic and abiotic challenges. In this work, we used an anti-K-ε-GG antibody for immunoaffinity enrichment and high-resolution mass spectrometry to quantify protein ubiquitination in maize seedlings with and without heat treatment. In total, 3492 Lys ubiquitination (Kub) sites were identified in 1780 proteins. Among these, 3030 (86.8%) Kub sites in 1519 proteins were identified as up-regulated targets, while 434 (12.4%) Kub sites in 314 proteins were identified as down-regulated targets. 

**Figure 5:**
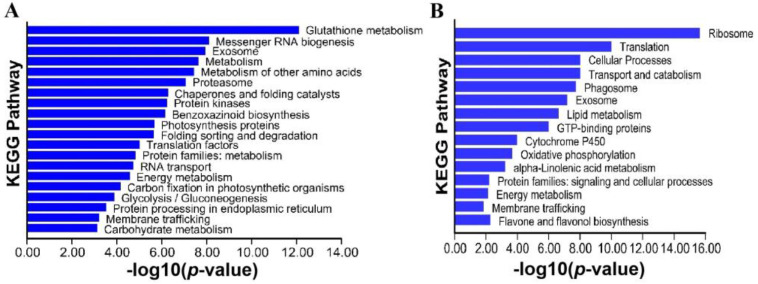
Pathway analyses of ubiquitylome: (**A**) KEGG pathways for up−regulated Kub proteins; and (**B**) KEGG pathways for down−regulated Kub proteins.

To gain a global understanding of the functions of the identified ubiquitinated targets, KEGG pathway analysis was performed on the up-regulated Kub proteins. The glutathione metabolism pathway was found to be most significantly enriched. The status of glutathione is a marker for oxidative stress, initiated by increased intracellular H_2_O_2_ content. A total of 35 components in the glutathione metabolism pathway were detected in the up-regulated Kub sites, indicating that heat shock might affect ROS levels through protein ubiquitination. Proteins involved in the proteasome, chaperones and folding catalysts, folding, sorting and degradation, and protein processing in the endoplasmic reticulum were also significantly enriched in the up-regulated Kub proteins. These proteins all point to protein processing and degradation pathways, suggesting that heat stress led to the ubiquitination of a constellation of proteins to initiate UPR and ERAD. Other up-regulated Kub proteins included those related to messenger RNA biogenesis, RNA transport, protein kinases, and photosynthesis (Figure 5A). KEGG pathways enriched with respect to the down-regulated Kub proteins were involved in ribosome biogenesis, translation, cellular processes, transport and catabolism, phagosome, exosome, lipid metabolism, and GTP−binding (Figure 5B). It is interesting to note that exosome, energy metabolism, and membrane trafficking pathways were present for both up- and down-regulated Kub proteins. Exosomes contain various molecules and play important roles in protein quality control and cell–cell communication. Trafficking proteins play a role in the delivery of newly synthesized proteins from the endoplasmic reticulum to their final destination, and play pivotal roles in the rapid response to environmental stimuli such as heat stress. The appearance of these two pathways in both up- and down-regulated Kub proteins imply a complicated mechanism of regulation through protein ubiquitination.

### 3.5. Analysis of Ubiquitination Sites of Lysine

**Figure 6:**
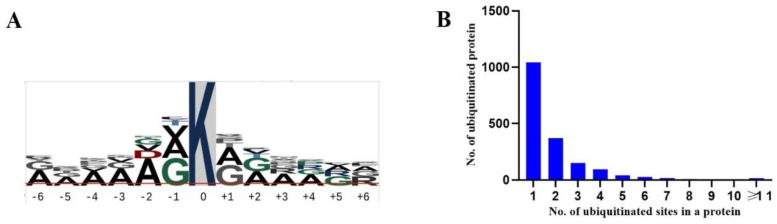
Consensus Kub motif and ubiquitination frequency analyses: (**A**) Amino acid preference around Kub sites; and (**B**) the number of Kub sites per protein.

To understand the sequence characteristics of the 3492 Kub peptides identified in maize, the p-logo program (https://plogo.uconn.edu/, accessed on November 2022) was used to analyze the position-specific amino acid residues around ubiquitinated lysines. A total of 13 amino acids, including 6 upstream and 6 downstream of the ubiquitinated lysines, were subjected to the analysis. The results showed that alanine (A) and glycine (G) were highly enriched on both sides of the ubiquitinated lysine (Figure 6A). At other positions, A was also the most frequently present residue. The amino acid preference for A and G around Kub sites indicates that a small or neutral residue might be required for such a modification. Lysine is a necessary amino acid widely distributed in plant proteins. To assess the ubiquitination frequency of lysine in different proteins, we calculated the number of Kub sites in each protein, and found that most proteins contained only a single Kub site (58.6%), while 20.8%, 8.5%, 5.3%, and 2.3% proteins contained 2, 3, 4, and 5 Kub sites, respectively, and less than 4.5% proteins had more than 6 Kub sites.

### 3.6. ERAD Components Were Widely Ubiquitinated after Heat Stress

**Figure 7:**
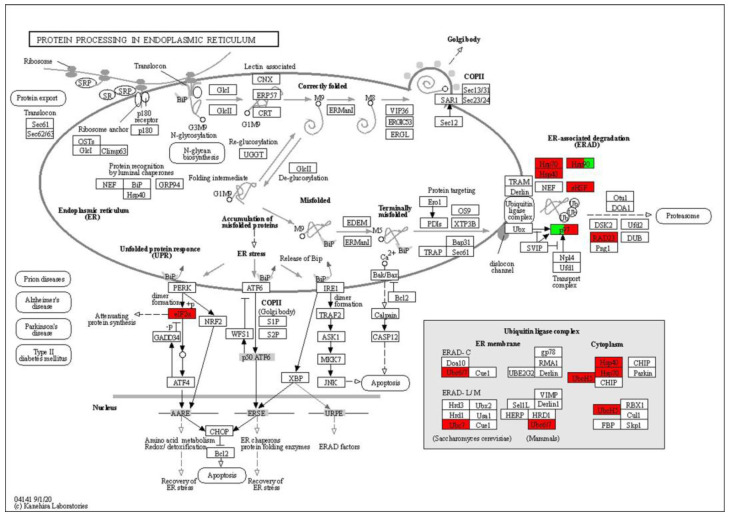
Ubiquitinated proteins of maize in the ER protein process pathway. Ubiquitinated proteins are highlighted in red or green: red indicates up−regulated Kub proteins, while green indicates down−regulated Kub proteins.

Based on KEGG analyses of the up- and down-regulated Kub proteins, we found that a number of proteins were related to the ER-associated protein processing pathway. This prompted us to examine whether one or more functional modules in this pathway were specifically ubiquitinated. The ER-associated protein processing pathway contains many steps, including protein translocation, translation on rough ER, protein glycosylation, folding, ERAD, and so on, as shown in the map in Figure 7. We searched for homologs of all of the maize proteins in this pathway against our ubiquitylome data, and mirrored them onto this map. Interestingly, almost all of them were related to the ERAD pathway, and most were up-regulated by heat stress. These included members of p97 proteins, Hsp70, sHSP, Ubc6/7, UbcH5, and RAD23 homologs.

Heat-shock proteins belong to the molecular chaperone family, which can bind unfolded or misfolded proteins to promote correct folding. Four HSP70 proteins were found to be hyper-ubiquitinated by heat stress. Among them, Zm00001d012420 was ubiquitylated at seven different lysines, with a maximum fold change of 58 compared to non-treated seedlings. One HSP40 (Zm00001d052855) and three HSP90 (Zm00001d052855, Zm00001d024903, and Zm00001d006008) proteins were also hyper-ubiquitinated. When plants are subjected to heat-shock stress, small heat-shock proteins (sHSPs) move to the outside of the plasma membrane to prevent the denaturation of membrane proteins and improve the stability of cell membranes. A total of 10 sHSPs were hyper-ubiquitinated to various degrees after heat treatment. In the ERAD machinery, the main driving force to extract ubiquitinated membrane proteins and to retro-translocate the luminal ERAD substrates is mediated by the AAA+ ATPase p97 (known as Cdc48 or VCP in humans). A maize p97 homolog, Zm00001d048409, was found to be hyper-ubiquitinated with a fold change of 3.36 after heat treatment. In addition, ubiquitin-conjugating enzyme (E2) UbcH5 homologs (Zm00001d012513 and Zm00001d043998) and Ubc6/7 homologs (Zm00001d028894, Zm00001d012421, Zm00001d010534, and Zm00001d042929), as well as a ubiquitin-like (UbL) domain protein (Zm00001d045665), were also hyper-ubiquitinated by heat stress. These chaperones and ubiquitination complex function to facilitate the degradation of misfolded proteins on the ER. They were differentially ubiquitinated after heat stress, suggesting feedback regulation of ERAD components by protein ubiquitination.

## 4. Discussion

High temperature severely affects plant growth and causes damage to cells in multiple aspects, including membrane integrity, protein activity, phytohormone signaling, oxidation–reduction balance, photosynthesis, and energy metabolism. Accordingly, plants have evolved various strategies to reduce the threat of heat stress. As the largest organelle in the cell, the ER plays vital roles in membrane and secreted protein translation, translocation, glycosylation, and folding. When plants are faced with heat stress, the need for protein processing exceeds the folding capacity of the ER, leading to ER stress. The abundant misfolded proteins accumulated in the ER trigger a series of cellular responses. HSR occurs in the cytosol to rapidly induce the transcription of several chaperone genes to expand the folding ability of the ER. At the same time, ER stress triggers UPR in the ER by peptide cleavage of bZIP28 or alternative splicing of *bZIP60* RNA. In addition to enhancing the folding capacity, unfolded proteins can also be cleared through the ERAD pathway. Several studies have assessed the proteome and ubiquitylome after heat stress; however, many proteins dynamically associate with the ER and co-exist in the cytosol and ER. Thus, the protein abundance and ubiquitination status in total cell extracts may not exactly reflect those in the ER. Considering the above, we isolated microsome fractions from maize seedlings with and without heat treatment, then conducted proteomic quantification and global ubiquitination analyses.

The microsomes were highly enriched for the ER fraction. Three antibodies were used to assess the purity of the microsome fraction: HSP90, a heat-shock protein that is localized in the cytosol; NADPH4, a mitochondrion protein; and HSC70, an ER lumen-localized heat-shock protein. Only HSC70 could be easily detected in the microsome fraction. Although we did not test other membrane organelles, such as the Golgi body and endosome, the fractions were largely free from cytosolic contamination and were highly enriched for the ER.

Integrity analyses of RNAseq and proteomics data indicated that 16 HSP genes were simultaneously up-regulated at the RNA and protein levels (Figure 4B). HSPs are among the most important targets of HSR and UPR. Heat stress causes the accumulation of misfolded proteins in the ER. To facilitate their correct folding, cells must produce large amounts of chaperones (e.g., HSPs) in a short time. The coordinated up-regulation of these HSP genes at the RNA and protein levels indicated that these genes were mainly regulated at the transcription level. This is in line with the mechanisms of HSR and UPR, both of which trigger the transcription of HSP genes (although they employ distinct strategies for this purpose). In contrast, a number of genes were also up-regulated at the protein level without changes at the RNA level. Several RNA binding proteins and E3 ligase-encoding genes were found in this group. The expressions of these genes are regulated by translational regulation. Through this manner of regulation, cells directly mobilized the stored RNAs to synthesize proteins without de novo RNA transcription under heat stress conditions. Translational regulation facilitates the prompt response of cells to environmental stimuli by bypassing the additional steps of RNA transcription, including recruiting transcription factors, RNA processing, and export from the nucleus. 

Global ubiquitination analysis revealed that Ala or Gly are preferred amino acids upstream and downstream of the Kub sites. We found that Ala was also preferred for other positions around the Kub sites. Both Ala and Gly are small in size and neutral in charge; the appearance of Ala or Gly around Kub sites possibly provides the motif with better flexibility and easier access to ubiquitin and E3 proteins. Interestingly, ERAD proteins were enriched in the ubiquitinated targets. ERAD is the security machinery of the cell for avoiding the detrimental effects of the accumulation of misfolded proteins. When the cell fails to repair the incorrect conformation, despite its best efforts, these misfolded proteins undergo degradation through the ERAD system. To this end, the proteins must be recognized and subjected to ubiquitin ligation. In addition to E1, E2, and E3 ligases on the ER, chaperones are also required to keep proteins in the appropriate conformations during this process. As 26S proteasomes are located in the cytosol, the ubiquitinated proteins must be retro-translocated from the ER to the cytosol for degradation. We found that proteins at almost every step of ERAD were ubiquitinated, including multiple HSP chaperones, E2 ligase, and AAA+ ATPase. As a ubiquitination-based protein degradation system, ERAD itself was also under the surveillance of ubiquitination, indicating that the activity of ERAD is subject to feedback regulation mediated by protein degradation. The mechanisms and components responsible for such feedback still require further investigation.

## 5. Conclusions

The ER is important for protecting plants against the damages caused by extreme heat, as ER proteins undergo dramatic changes in translation and post-translational modifications under heat stress. Our results demonstrated that the abundances of 2383 proteins and the ubiquitination status of 1780 proteins in the ER were significantly changed under heat treatment. ERAD plays a central role in the clearance of misfolded proteins from the ER, and the ubiquitination status of many ERAD components was obviously altered in response to heat stress.

## Data Availability

The RNAseq data generated in this study were deposited at NCBI BioProject under the accession number PRJNA922509. The proteosome and ubiquitylome data were submitted to ProteomeXchange via the PRIDE database under the accession number PXD039418.

## Supplementary Materials

The following supporting information can be downloaded at: https://www.mdpi.com/article/10.3390/genes14030749/s1, Table S1: Gene IDs and primers for Real-Time PCR.
