# Supplementary material for: Proteome and Ubiquitylome Analyses of Maize Endoplasmic Reticulum under Heat Stress
Source: Genes (Basel). 2023 Mar 19;14(3):749. doi: 10.3390/genes14030749 (PMC10047965; doi:10.3390/genes14030749)
Supplement: Supplementary file 1 [file genes-14-00749-s001.zip › genes-2236080-supplementary.pdf]

Table S1. Gene IDs and primers for Real-Time PCR.

Gene Name	Gene ID	Forward	Reverse
ZmHSP26	Zm00001d028408	GAGCACAAGAAGGAGGAGGG	CCTGCACGTCGATGACCTT
ZmCRT1a	Zm00001d005460	AGTCCGAGTGGAAAAAGGACG	AAGTTGTGTCCCCACCGAAT
ZmPDI1	Zm00001d049099	AGGCTACCCGACCTTGTACT	GATGCTCACAGCTCGTCCTT
ZmbZIP60	Zm00001d046718	GCAGAGTGCCGTCGCCTCAGCTAC	GGCAGGGTTTCCGTGAGTAC
ZmHsf-01	Zm00001d027757	GAGAACCTGGCGCTCAACAT	CGCCCTTCATCCCTTCGT
ZmHsf-03	Zm00001d031736	GAGCAGGTGCTGTCGTCAAG	TTGTTGCACAGCTTCTTCATCTG
ZmHsf-04	Zm00001d032923	AGCAGCAAGACAAGAGGAAGGA	TGTTCGCTTTCCCCATCACT
ZmHsf-25	Zm00001d026094	GAGGACAACGACGAGGAACAA	AGCGTCAGCACGTCCAGATC
ZmbZIP17	Zm00001d007042	GAAGCATGTATAGGGAGGAGG	TCTTGAGTGAAGTTCTGTGACG
TUB	Zm00001d046996	CTACCTCACGGCATCTGCTATGT	GTCACACACACTCGACTTCACG
